# Patterns of cell proliferation and apoptosis by topographic region in normal Bos taurus vs. Bos indicus crossbreeds bovine placentae during pregnancy

**DOI:** 10.1186/1477-7827-7-25

**Published:** 2009-03-30

**Authors:** Patricia R Facciotti, Rose EG Rici, Durvanei A Maria, Marcelo Bertolini, Carlos E Ambrósio, Maria A Miglino

**Affiliations:** 1 Department of Surgery of the São Paulo University, Faculty of Veterinary Medicine and Animal Science, São Paulo, SP, 05508-270, Brazil; 2 Biochemical and Biophysics Laboratory, Butantan Institute, São Paulo, 05503-900, Brazil; 3 UDESC – Santa Catarina State University, School of Veterinary Medicine, Lages, Santa Catarina, 88520-000, Brazil; 4 Department of Basic Science/FZEA, São Paulo University, Faculty of Veterinary Medicine, Pirassununga, SP, 13635-900, Brazil

## Abstract

**Background:**

Placental and fetal growth requires high rates of cellular turnover and differentiation, which contributes to conceptus development. The trophoblast has unique properties and a wide range of metabolic, endocrine and angiogenic functions, but the proliferative profile of the bovine placenta characterized by flow cytometry analysis and its role in fetal development are currently uncharacterized. Complete understanding of placental apoptotic and proliferative rates may be relevant to development, especially if related to the pathogenesis of pregnancy losses and placental abnormalities.

**Methods:**

In this study, the proliferation activity and apoptosis in different regions of normal bovine placenta (central and boundary regions of placentomes, placentomal fusion, microplacentomes, and interplacentomal regions), from distinct gestation periods (Days 70 to 290 of pregnancy), were analyzed by flow cytometry.

**Results:**

Our results indicated that microplacentomes presented a lower number of apoptotic cells throughout pregnancy, with a higher proliferative activity by the end of gestation, suggesting that such structures do not contribute significantly to normal of placental functions and conceptus development during pregnancy. The placentome edges revealed a higher number of apoptotic cells from Day 170 on, which suggests that placentome detachment may well initiate in this region.

**Conclusion:**

Variations involving proliferation and apoptotic rates may influence placental maturation and detachment, compromising placental functions and leading to fetal stress, abnormalities in development and abortion, as frequently seen in bovine pregnancies from in vitro fertilization and cloning procedures. Our findings describing the pattern of cell proliferation and apoptosis in normal bovine pregnancies may be useful for unraveling some of the developmental deviations seen in nature and after in vitro embryo manipulations.

## Background

In ruminants, the placenta is classified as cotyledonary on the basis of its gross anatomical features. The immediate feto-maternal contact in the bovine placenta is restricted to multiple discrete structures called placentomes, consisted of the maternal part, the caruncle, and the fetal part, the cotyledon. In early gestation, caruncles and cotyledons are not connected yet, without formation of placentomes [1-3]. Placentome formation can be considered a gradual process that takes place immediately after pregnancy recognition, being fairly completed by the end of organogenesis [4]. Once established, bovine placentomes grow continuously throughout pregnancy. However, placentome growth varies considerably in the course of gestation. Between days 100–160, placentomes show an accelerated growth pattern, reaching its plateau by around day 199. After this phase, placentome growth decreases progressively [5] and is minimal in late gestation [1]. The growth of the foetal and maternal structures that compose the placentomes are different in feature and are differentially regulated, and the proportion of foetal to maternal tissue is specifically controlled by unknown factors that do not act consistently throughout gestation [6].

Coalescence between two or more neighboring caruncles was observed in Egyptian buffalo cows [7]. Those authors observed that the unusual lobulated shape of caruncles may result from the fusion of four or more adjacent ones. Fusion between the neighboring caruncles occurred only in the fibrillar region and thus deep fissures are found on their free convex surface. Most of the caruncles were found at the antimesometrial region of the uterine horn varying in size, number and shape during all pregnancy phases [8,9]. The placentomal fusion presence was also observed in cloned cattle term placenta [10] and was observed a high number of fusions resulting in a increase of placentome size and decrease in numbers.

Bovine normal placentae also presented microplancetomes called accessories placentomes, which have a diameter less than 1.0 cm and with different numbers both found at the extremities of pregnant or not horns. [1,11].

In the formation of mammalian placenta, many factors must interact in a precise manner to a normal placental development. The uterus increases in size to accommodate the bovine fetus, with the size of the uterus not influencing fetal size [12]. However, maternal uterine environment influences fetal growth and those influences may be mediated, in part, by growth and function of caruncular tissues [13].

Placental growth is complex and influenced by many factors, and it is dependent upon a delicate balance between cell proliferation, differentiation, and death [14]. Considering the distinct macroscopic and functional regions existent at the bovine placenta, diverse patterns of cell proliferation and apoptosis are expected in those regions. A few studies have evaluated the proliferative activity in bovine placentomes [15,16] or the trophoblastic cell density at the end of gestation in cattle [17], but the spatial and temporal profiles for cell proliferation and apoptosis characterized by flow cytometry in the bovine placenta are yet to be described. The understanding of such profiles may assist in sorting out mechanisms leading to placental and fetal abnormalities, especially those associated with certain in vitro embryo manipulations, such as in vitro fertilization (IVF) and cloning by somatic cell nuclear transfer (SCNT) procedures [4,18]. Despite their wide use around the world today, such embryo technologies are associated with pre- and postnatal abnormalities, including increased rates of pregnancy losses, hydrops of the fetal membranes, prolonged gestation, diminished signs of parturition, dystocia, and birth of large calves with lower postnatal survival [4]. Placental tissues from cloned bovine gestations had shown more proliferation activity and a reduced rate of apoptosis in the central region of the placentomes and interplacentomal areas that may be associated with the uncommon abnormalities after in vitro embryo manipulations [19]. The appearance of such a diverse range of abnormalities does compromise the practical application of those technologies [20], but also ensures that novel knowledge is gained in mammalian developmental biology, as for one to know the abnormal, the characterization of the normalcy is essential. Therefore, the aim of this study was to characterize normal placenta development during gestation in cattle, describing cell proliferation and apoptotic profiles in distinct topographic regions of the placenta based on the DNA content analysis by flow cytometry. The choice of each placental region was based on different areas in the same placentome (central and edges), interplacentomal areas, placentomal fusion and microplacentome, which show different cell activities during placentation, however with low number of studies in this field.

## Methods

### Animals and tissue sampling

A total of 28 bovine placentas (Bos taurus vs. Bos indicus crossbreeds) at different gestational phases were collected at a local abattoir. The uterus of each animal was cut open, the fetus removed, and the crown-rump length used to estimate the stage of gestation [21,22]. Placentae were divided into four groups of 7 animals each: group I (70 to 120 days), group II (121 to 170 days), group III (171 to 220 days), and group IV (221 to 290 days).

**Figure 1:**
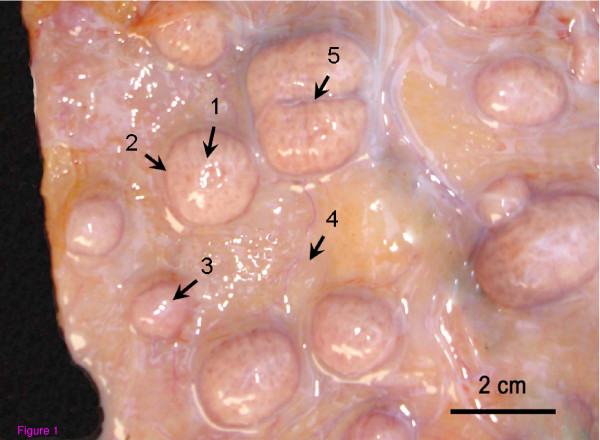
**Representation of the distinc placental regions from which tissue samples were collected for flow cytometry analysis**. (1) Central region of the placentome, (2) Placentome edges, (3) Microplacentome, (4) Interplacentomal region, (5) Placentomal fusion. Bovine pregnant tract at 122 days of gestation.

Different regions of each placentae were collected, namely the central region of the placentome, the placentome edges, interplacentomal areas, placentomal fusion, and microplacentomes (≤1.0 cm), as illustrated in Figure 1. Two placental tissue pieces, collected from each placental region from each animal, were gently excised and cut into numerous small fragments of around 0.5 cm, to be immediately plunged into liquid nitrogen and subsequently used for flow cytometry analysis. Due the anatomical particularities of interplacentomal region during the mechanic dissection, myometrium was gently extracted and kept the total tissue with fetal and maternal epithelia, also fetal mesenchyme for flow citometry analyses. All placental tissue pieces contained both maternal and foetal tissue, including connective tissue and uterine and trophoblastic epithelia. Therefore, the flow cytometric analysis was performed on a cell population composed largely of epithelial cells, with a rare proportion of maternal stroma and foetal mesenchyme, such as fibroblasts and endothelial cells. The uterine subepithelial cell in bovines is richly vascularized and composed by a great amount of fibrocytes [23], which are in quiescent metabolic status [24], and not significantly interfering on the proportion of cells in the proliferation (S, G2/M) or in apoptosis.

### Flow cytometry analysis – DNA content and cell cycle distribution

Fragments stored in liquid nitrogen were placed in citrate buffer, pH 7.6, and processed according to previous experiments [25], being subsequently filtered through a 30 μm mesh (Spectra Mesh Nylon Filters, Sigma Chemical Co., St. Louis, MO, USA) to separate proteins (a large component of the tissue stroma, or connective tissue) and cell debris, composed of blood cells, degenerating/degenerated cells and adipose tissue. Samples were incubated in 30 mg/mL trypsin (Sigma Chemical Co.) for 10 min at room temperature. Then, 5 mg/mL trypsin inhibitor (Sigma Chemical Co.) and 0.1 mg/mL of ribonuclease A (Sigma Chemical Co.) were added for 10 min at room temperature (for optimal DNA resolution), and 415 μg/mL propidium iodide (Sigma Chemical Co.) was added 15 min before the flow cytometry analysis. At least 10,000 events were acquired using Cell Quest Software (Beckton Dickinson, San Jose, CA, USA). The DNA content was measured using a FACScalibur Flow Cytometer (Beckton Dickinson) equipped with an air-coupled 15-mW, 488-nm argon ion laser. The percentage of cells in each phase of the cell cycle, namely apoptosis (sub-G1, DNA fragmentation), G0/G1 (quiescent cells), synthesis (S), and G2/M (proliferative phase) was determined with ModFit Software analyses (Beckton Dickinson).

Data from cytometry experiments were subjected to one-way ANOVA followed by pairwise multiple comparison procedures (Student-Newman-Keuls method) using GraphPad Instat 3.0 software.

## Results

The cell cycle analysis was performed in placental tissues throughout pregnancy, starting at a time when placentomes are already well formed. The percentage of cells in each phase of the cell cycle for each topographic region of the bovine placenta by group is displayed in Table 1.

**Table 1:** Distribution of the cell cycle phases (in %) in distinct topographic regions of placentae at different stages of gestation in cattle

		Placental region					
		Placentome					
Cell cycle phase	Groups: Days in gestation	Total*	Central region	Edges	Interplacentomal region	Microplacentomes	Placentomal fusion
G2/M	G-I: 70 to 120	20.0 ± 11.4^a, b^	16.7 ± 7.0^a, b^	23.4 ± 15.9^a^	23.6 ± 3.1^a^	8.9 ± 6.2^b, B^	7.7 ± 4.3^b^
	G-II: 121 to 170	20.3 ± 10.2^a^	18.9 ± 9.3^a^	21.7 ± 11.1^a^	18.3 ± 9.3^a^	7.7 ± 2.1^b, B^	5.4 ± 3.4^b^
	G-III: 171 to 220	21.6 ± 12.1^a, b^	19.6 ± 13.7^a, b^	23.6 ± 10.6^a^	17.6 ± 9.4^a, b^	13.1 ± 12.4^a, b^	7.3 ± 4.1^b^
	G-IV: 221 to 290	18.8 ± 7.7	18.6 ± 5.9	19.0 ± 9.6	18.5 ± 6.5	21.8 ± 8.4^a^	12.1 ± 11.3
S	G-I: 70 to 120	5.6 ± 3.5	5.7 ± 3.4	5.5 ± 3.6	5.6 ± 2.5	3.7 ± 3.1	3.1 ± 0.3
	G-II: 121 to 170	4.4 ± 0.9	4.6 ± 0.9	4.1 ± 1.1	3.0 ± 1.4	3.2 ± 0.7	3.4 ± 1.1
	G-III: 171 to 220	7.4 ± 4.6	10.2 ± 7.4	4.5 ± 1.8	5.0 ± 4.2	5.5 ± 4.6	3.6 ± 1.6
	G-IV: 221 to 290	5.6 ± 3.3	8.2 ± 4.9	3.0 ± 1.8	3.7 ± 2.6	4.4 ± 3.9	3.0 ± 1.5
G0/G1	G-I: 70 to 120	63.7 ± 16.3^a, b^	67.0 ± 13.8^a, b^	60.4 ± 18.9^a^	57.0 ± 9.1^a^	84.3 ± 11.8^b^	80.5 ± 10.9^b^
	G-II: 121 to 170	60.5 ± 18.1^a^	64.6 ± 13.8^a^	56.5 ± 22.3^a^	64.3 ± 18.3^a, b^	86.4 ± 2.0^b^	87.7 ± 3.9^b^
	G-III: 171 to 220	54.2 ± 20.7^a^	62.2 ± 18.1^a, b^	46.2 ± 23.4^a^	67.6 ± 15.7^a, b^	78.8 ± 18.5^b^	83.0 ± 7.1^b^
	G-IV: 221 to 290	56.1 ± 17.8	62.5 ± 10.0	49.8 ± 25.7	65.1 ± 15.8	71.2 ± 11.0	73.5 ± 22.1
Apoptosis	G-I: 70 to 120	10.7 ± 8.5^a^	10.6 ± 8.5^a^	10.7 ± 8.5^a^	13.7 ± 9.8^a^	3.0 ± 2.6^b^	8.6 ± 8.5^a^
	G-II: 121 to 170	14.8 ± 8.9^a^	12.0 ± 5.7^a, b^	17.7 ± 13.3^a^	14.4 ± 8.9^a^	2.7 ± 0.4^b^	3.5 ± 0.7^b^
	G-III: 171 to 220	16.9 ± 7.9^a, b^	8.0 ± 3.7^b^	25.7 ± 13.9^a^	9.7 ± 8.2^b^	2.7 ± 2.1^b^	6.1 ± 3.3^b^
	G-IV: 221 to 290	19.5 ± 8.3^a, b^	10.7 ± 4.7^b^	28.2 ± 17.2^a^	12.7 ± 10.3^b^	2.6 ± 1.1^b^	11.4 ± 9.4^b^

^a-e ^Values (mean ± SD) in the same row with different superscripts differ (P < 0.05).

^A-E ^Values (mean ± SD) in the same column with different superscripts differ (P < 0.05).

* Total means: central plus edges region of placentome

**Figure 2:**
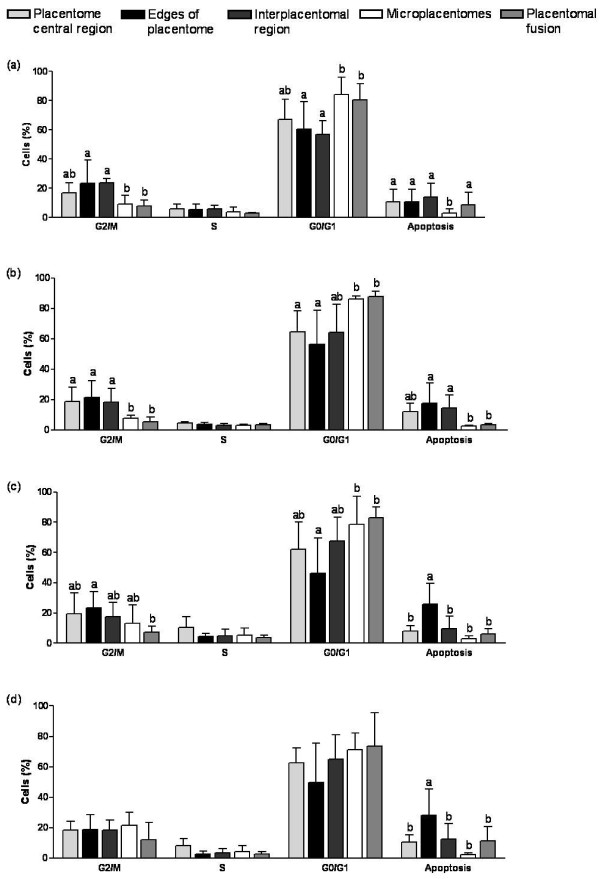
**Distribution of the phases of the cell cycle (G2/M, S, G0/G1, apoptosis) in distinct placental regions**. (a) Group I (Days 70 to 120 of gestation), (b) Group II (Days 121 to 170 of gestation), (c) Group III (Days 171 to 220 of gestation), (d) Group IV (Days 221 to 270 of gestation).^a-b ^Bars with different superscripts differ; P < 0.05.

In group I, a lower number of G2/M cells (P < 0.05) and a higher number of G0/G1 cells (P < 0.05) were observed in microplacentomes and placentomal fusions in comparison with the interplacentomal region and the edges of the placentomes, indicating a lower proliferative activity in the former structures, along with a lower cell death rate. In this gestational group, the incidence of apoptotic cells were lower in microplacentomes (P < 0.05) in comparison with the other placental regions (Figure 2a).

Significant variations remained on microplacentomes and placentomal fusions on Group II, as shown in Figure 2b. Those structures remained with a lower number of cells in proliferative activity in relation to the other regions, demonstrating significantly lower values for cells in G2/M phase (P < 0.05) and higher number of cells in G0/G1 phase (P < 0.01). The number of apoptotic cells was also lower in microplacentomes in relation to the other regions (P < 0.05), except the central region of placentome. However, a lower number of apoptotic cells were also observed in placentomal fusions in Group II, indicating a lower cell death rate.

In Group III, the number of G0/G1 cells differed significantly in the edges of placentomes (P < 0.05) and in total placentome (central region plus edges) in relation to microplacentomes and placentomal fusions (Figure 2c). The number of G2/M cells was significantly higher in the edges of placentomes (P < 0.01) in relation to placentomal fusions. Surprisingly, there were a significantly higher number of apoptotic cells in those boundary areas in groups III and IV (P < 0.001), except the total placentome related to central region plus edges areas.

Significant variations were observed in numbers of apoptotic cells in Group IV, as shown Figure 2d. The incidence of apoptotic cells was significantly higher in the placentome edges (P < 0.001) in relation to the other regions, except the total placentome (central region + edges).

There were no significant differences in proliferation activity between the interplacentomal region and the central region of placentome, those regions revealed similar values in both cell cycle phases and gestational groups.

## Discussion

Our findings by flow cytometric analysis indicated variations in proliferation and apoptosis in different placental regions at each gestational phase. Analyzing the cell cycle phases were found a high standard deviation among the samples, probably due to the high variation of chorionic:uterine cells proportion. However, we aimed to determine the proliferative activity and apoptosis in all studied placental regions like as maternal and fetal tissue and the high standard deviation were expected.

By analyzing the total placentomes, area, i.e., cells from the edge and from the central placentome region, we observed a proliferative activity that was inversely proportional to the apoptotic rate throughout gestation, which was not detected when regions were analyzed separately. According to a previous report [16], apoptosis reflects the beginning of tissue regeneration and continuing histiotrophic nutrition to the fetus, which consists in phagocytosis of maternal apoptotic epithelial cells. Such variations are considered part of the placental maturation process, which can be a requirement to materno-fetal detachment and fetal membranes release [1,16]. Such variations were not detected in term placentas from clone cattle. Using flow cytometry, [19] showed more proliferation activity and a reduced rate of apoptosis in the central region of the placentomes and interplacentomal areas. Such phenomena may be associated with faulty placentation in early pregnancy, placental insufficiency or even a lack of placental and/or fetal maturation towards the end of pregnancy.

When placentome's edges were analyzed separately from the central region of the placentome, a premature increase in apoptotic cells was detected, with a significantly higher number of apoptotic cells than in the other regions as pregnancy progressed, well observed in Groups III and IV. According to [16], the large number of apoptotic cells by the end of gestation occurs as a mechanism associated with maternal-fetal detachment. Therefore, the high number of apoptotic cells in the placentome's edge from 170 days on suggests this region to be the point of initiation of placentomal detachment in cattle. In this context, the edges of placentome may play an important role in placental maturation and release. These results suggest that placentomal detachment occurs in a centripetal way; therefore, the placentomal maturation occurs from the edges to the center of the placentomes.

TUNEL procedure and Ki-67 proliferation maker demonstrated proliferation not to be the main factor responsible for growth in interplacentomal uterine wall, including adherent fetal membranes, which predominantly should be achieved by hypertrophy [26]. Findings from that study gives support to ours for the interplacentomal region, which showed no significant variations during pregnancy, suggesting that proliferation is of minor importance for tissue homeostasis in the interplacentomal region.

In this study, microplacentomes showed different proliferative patterns throughout pregnancy. The G2/M cells increased in numbers throughout gestation, with significative variations in groups I and II in relation to group IV, suggesting cell cycle progression from a quiescent to a proliferative state. On the other hand, the proportion of apoptotic cells in microplacentomes remained low in all gestational periods, with percentile values being significantly lower than the other studied regions in Group I; the total placentome (central region + edges), the placentome's edges and interplacentomal region in Group II; and the placentome's edges in Groups III e IV. Such results suggest microplacentomes to have a slower proliferation activity in Groups I and II, which could be linked to its limited size in bovine placenta. Apparently, such structures remain at a quiescent state in group I, with low tissue plasticity or remodeling, not following the pattern of maturation and growth seen in normal placentomes. Most likely, the observations above are related to the relatively small role of those structures, with microplacentomes not contributing significantly to normal of placental functions and conceptus development during pregnancy.

Placentomal fusion showed a lower proliferative activity in Groups I and II compared to the other regions, which was similar to microplacentomes. The number of proliferative cells increases on Group IV, however, the number of apoptotic cells also increases in the same period, demonstrating a balance between proliferative and apoptotic cells number in placentomal fusion. The region of fusion is composed by endometrial stromal axis [8], formed by cells in a balance/equilibrium state of proliferative activity, suggesting maternal stromal axis have a functional role in physiological maintenance of gestation, however, a little contribution to maturation and placental release.

Abnormal placental development has been implicated as the main factor limiting the success in ruminant pregnancies derived from IVF and cloning procedures by SCNT [4,18]. Recent studies have described structural and microvascular architecture distinctions in bovine IVF and clone placentae [10,27], with the description of increased number of functional and accessory microcotyledons, dilated caruncular crypts accommodating more than one primary villous with a lack of dense complexes of capillary loops and sinusoidal dilations, abnormal vascularization, tissue remodeling, differentiation and maturation of placental tissue in mid- to late pregnancy. In that period, any alteration in placenta development may affect fetal development and the success of gestation [28], with disturbances in programmed cell death in placenta seeming to be associated with an abnormal pregnancy outcome [29-32]. Taken together, the complete understanding of placental apoptotic and proliferative profiles at distinct placental tissue regions during gestation will shed light to unknown physiological processes related to conceptus development, as some regions may be potentially responsible, at different degrees of influence, to distinct modulation patterns in fetal growth and nutrition throughout pregnancy. In that regard, results from the present study using non-manipulated and crossbreed bovine gestations indicated that proliferation and apoptosis exhibit patterns that are specific for the individual placental areas during gestation and at term in normal, non-manipulated pregnancies in cattle.

## Competing interests

The authors declare that they have no competing interests.

## Authors' contributions

PRF, REGR, DAM and MAM collected the materials, established the pregnancy groups, performed the cytometry analysis, carried out the experiment and wrote major parts of the manuscript. MB wrote parts of the manuscript regarding the physiology of reproduction. CEA, MB and MAM reviewed the manuscript and the quality of the written English. All authors read and approved the final manuscript.
